# Medical Jurisprudence

**Published:** 1850-07

**Authors:** 


### REVIEWS.

---

Art. V.—*Medical Jurisprudence.* By Alfred S. Taylor, F. R. S., member of the Royal College of Surgeons; and Professor of Medical Jurisprudence and Chemistry in Guy's Hospital. Second American, from the third London edition. With notes and additions by R. Eglesfeld Griffith, M. D., &c. Philadelphia: Lea & Blanchard. 1850.

The science of medical jurisprudence has accumulated a great many facts, corrected numerous errors of opinion, and securely established many important principles, within the past few years. There is no department of medicine that has traveled with a steadier pace; there is none that has gathered more valuable fruit. But, although it has thus improved, much yet remains to be done. The hopes of phrenologists have not been fulfilled, for many of the ardent votaries of that science, thought a few years ago, that it would take a prominent place in medical jurisprudence, in determining how far criminals were accountable beings. It was supposed that phrenology would be able to make the exact boundary lines, between general insanity and monomania, and between monomania and sanity. The courts of justice, however, could not be made to participate in the confidence of phrenologists in these matters, and the judicial murder of Dr. Baker, in this State, and the reprehensible conduct of the Lord Chief Baron, in the case of Miss Nottidge, of England, are mournful examples of the want of fixed data for ascertaining the boundary lines of sanity, and insanity. Indeed, in all matters of a medico-legal nature, the courts of England and America have remarkably loose notions, and there are no witnesses treated, generally, with the same degree of negligence, that those belonging to the medical profession usually receive. We have known juries to give verdicts against the medical evidence, even where the professional witnesses were unanimous. It is somewhat singular that upon that only ground, where law and medicine meet, the practitioners of the two sciences should entertain feelings and opinions diverse from each other. Medical witnesses are often looked upon by the lawyers in the case, very much as a bear is by a parcel of butcher boys—as something that may afford fine sport.

That a portion of this evil is founded on a vital error in the administration of justice we are well satisfied. Lord Brougham has laid down the atrocious maxim that a lawyer, in the management of a case, should know no being in the universe but his client; that he must make himself a part of his client, and do all things that can be done to acquit the criminal. Why a criminal lawyer may forget society; why he is privileged to set at naught all the recognised principles of right and of virtue, and may tower over every other citizen, and over all the institutions of government, we do not understand, nor do we believe that we could be made to understand these things. It is clearly the duty of a lawyer, as it is of every good citizen, to see that the laws do not commit an outrage upon a client, beyond that a lawyer has no more right to go than a citizen has. It is the prerogative of the court to secure a clear administration of justice, and to permit no one within its jurisdiction to obstruct or prevent that administration.

It seems to us that among other fruits of injustice and wrong growing out of these things, the maltreatment of medical witnesses by lawyers is conspicuous, and to th[illegible]

is to be attributed much of the indisposition of juries to rely on medical testimony. But if the court is well-informed on subjects of medical jurisprudence, and protects medical witnesses, who know what they are talking about, in the exercise of their duties, the administration of justice will be forwarded, and society will enjoy greater security than it does now.

The works of Christison and Guy have done a great deal towards furthering the laudable objects we have referred to, and the valuable book before us has ample capacity for aiding the good work. The first edition was published in 1844, and a third edition has been already called for. The author says: "In preparing this edition, no pains have been spared in order to keep the work on a level with the advanced state of science. The medical journals of the United Kingdom, as well as those of France, Germany, Italy, and America, have been searched for those newly-observed and accurately recorded facts, which are constantly increasing the stores of medical experience. A proper selection of these in their medico-legal relations has been made for this edition; and those legal cases have been added, which have given rise to new questions requiring the aid of a medical jurist for their solution."

Eight new chapters have been written, and many medico-legal observations and cases, have been introduced into this edition. "Chemical, microscopical, and pathological researches have each contributed within a recent period to the resources of the medical jurist; and the new aids to the detection of crime which have thus been brought to light, are here laid before the reader in a concise but, it is hoped, a practical form."

"The section on POISONING will be found to contain a description of many *new tests and processes*, as well as many additional facts connected with the *action of poisons* on the body."

"In the sections on WOUNDS, additions have been made on the following subjects: the medico-legal examination of wounds; physical, chemical, and microscopical evidence in reference to *blood-stains; on imputed or self-inflicted wounds;* medico-legal facts connected with the fatal results of *surgical operations; medical responsibility and malpraxis; death from entrance of air into the veins; rupture of the kidney and urinary bladder*; *spontaneous fractures; wounds from fire arms not loaded with ball; varieties of burns; injuries from melted metals;* and on *injuries (simulating wounds) caused by fire.*"

"In the section on INFANTICIDE, the reader will find additions to the chapters—on the death of the child *before respiration;* on the alteration in the *chemical composition of the lungs* by respiration; on marks of violence, and on the detection of *food in the stomach as signs of live birth;* additional observations on the *hydrostatic test;* on the newly discovered fallacies of the *docimasia circulationis*; *the congenital closure of the ductus arteriosus and foramen ovale;* and on the occurrence of the severe *accidental injuries* to children *during birth.*"

The chapters on PREGNANCY AND DELIVERY have been almost entirely re-written. Under these heads will be found additional cases and observations on the *plea of pregnancy*, and on the *signs of delivery* in the *dead* in cases of *criminal abortion.* Under this branch of the subject, the *new* views of physiologists regarding the evidence derivable from the presence of *corpora lutea* are also fully considered."

"Many additional facts have been recorded in reference to the medico-legal bearings of the *Cæsarean operation*; *tenancy by courtesy*, and age and minority. The chapter of LEGITIMACY has been considerably enlarged. The evidence given on some important trials of recent occurrence, has rendered it necessary to re-arrange the hitherto ascertained facts regarding *premature* and *protracted births*—

the determination of the period of *uterine life*, from the *development of the offspring*, as well as certain questions connected with PATERNITY. * * * In the section on INSANITY, there will be found, among the additions, the recent decision of the judges on *medical certificates*, with new cases and observations."

These quotations from the author's preface set forth the leading improvements in the third edition of this valuable work. The various departments of medical jurisprudence are admirably handled in this book; there is, indeed great fullness on all subjects, except on POISONS. The author felt himself authorized to abridge his remarks on that theme, because of his recent publication of a separate work on poisons. But even in the abridged form the reader will find an ample fund of information on all points "upon which medical opinions are most urgently required." Many NEW TESTS and PROCESSES are given, as well as additional facts connected with the *action of poisons* on the body."

Dr. Griffith has performed the duties of editor, with ability. His notes are very admirable and judicious.

We can cordially commend this work, as one admirably adapted to the wants of the medical and legal professions. The diligent student of such a work as this, may appear in a court of justice with confidence in his ability to shed light on the most intricate points.

The publishers have done themselves credit in the excellence of the execution of their part of this work.

